# NT1721, a novel epidithiodiketopiperazine, exhibits potent *in vitro* and *in vivo* efficacy against acute myeloid leukemia

**DOI:** 10.18632/oncotarget.13364

**Published:** 2016-11-15

**Authors:** Claudia M. Kowolik, Min Lin, Jun Xie, Larry E. Overman, David A. Horne

**Affiliations:** ^1^ Department of Molecular Medicine, City of Hope National Medical Center, Duarte, CA 91010; ^2^ Department of Chemistry, 1102 Natural Sciences II, University of California, Irvine, CA 92697-2025

**Keywords:** ETP, FLT3-ITD, DNMT1, BMI1, FLT3 ligand

## Abstract

Acute myeloid leukemia (AML) is an aggressive malignancy characterized by heterogeneous genetic and epigenetic changes in hematopoietic progenitors that lead to abnormal self-renewal and proliferation. Despite high initial remission rates, prognosis remains poor for most AML patients, especially for those harboring internal tandem duplication (ITD) mutations in the fms-related tyrosine kinase-3 (FLT3). Here, we report that a novel epidithiodiketopiperazine, NT1721, potently decreased the cell viability of FLT3-ITD^+^ AML cell lines, displaying IC_50_ values in the low nanomolar range, while leaving normal CD34^+^ bone marrow cells largely unaffected. The IC_50_ values for NT1721 were significantly lower than those for clinically used AML drugs (i.e. cytarabine, sorafenib) in all tested AML cell lines regardless of their FLT3 mutation status. Moreover, combinations of NT1721 with sorafenib or cytarabine showed better antileukemic effects than the single agents *in vitro*. Combining cytarabine with NT1721 also attenuated the cytarabine-induced FLT3 ligand surge that has been linked to resistance to tyrosine kinase inhibitors. Mechanistically, NT1721 depleted DNA methyltransferase 1 (DNMT1) protein levels, leading to the re-expression of silenced tumor suppressor genes and apoptosis induction. NT1721 concomitantly decreased the expression of EZH2 and BMI1, two genes that are associated with the maintenance of leukemic stem/progenitor cells. In a systemic FLT3-ITD^+^ AML mouse model, treatment with NT1721 reduced tumor burdens by > 95% compared to the control and significantly increased survival times. Taken together, our results suggest that NT1721 may represent a promising novel agent for the treatment of AML.

## INTRODUCTION

Acute myeloid leukemia (AML) is an aggressive, extremely heterogeneous cancer associated with genetic and epigenetic changes that result in increased self-renewal ability, proliferation and impaired differentiation of hematopoietic stem and progenitor cells [1]. It is the most common type of leukemia in adults, accounting for ∼30% of leukemias and > 40% of leukemia-related deaths [2]. The efficacy of the standard treatment, i.e. remission induction chemotherapy with an anthracycline/cytarabine combination followed by either consolidation chemotherapy or allogeneic hematopoietic stem cell transplantations, is limited; 10–40% of patients do not respond to induction therapy and 50–70% of patients achieving complete remission are expected to relapse within 3 years [3, 4]. The prognosis for AML patients with FLT3-ITD mutations is especially poor: FLT3-ITD mutations are detectable in ∼30% of AML patients and are associated with increased resistance to cytarabine, higher relapse rates and shorter median survival times [5–8]. FLT3-ITD mutations cause constitutive FLT3 activation, leading to aberrant signals that promote cell growth and inhibit apoptosis [2]. Recognition of FLT3 mutations as one of the key drivers of AML led to the development and clinical evaluation of several tyrosine kinase inhibitors (TKIs). Despite promising initial antileukemic activity of several FLT3 inhibitors in preclinical and clinical studies, none has been approved for routine clinical use in AML [9]. While recent studies show that adding FLT3 inhibitors to conventional chemotherapy can improve the clinical outcome [10, 11], responses to monotherapy with TKIs have only been incomplete or transient in the majority of patients treated with a single agent TKI due to rapidly developing drug resistance [1, 2, 6, 12]. Mechanisms of TKI resistance include the emergence of new mutations and bone marrow stroma-mediated protective effects, which result in cell cycle arrest rather than apoptosis of AML cells [12, 13]. Moreover, exposure to chemotherapy drugs such as cytarabine leads to a surge in FLT3 ligand (FLT3LG) expression in AML cells and stroma, which increases IC_50_ values for TKIs *in vitro* and attenuates the efficacy of TKIs *in vivo* [8, 14–16]. Furthermore, reports show that CD34^+^/CD38^−^ leukemia stem cells are highly drug resistant, which at least partially accounts for the high relapse rate in AML [17, 18]. Given the evident lack of effective treatment options for relapsed or refractory AML, there is urgent need to developnovel therapies for AML.

Epidithiodiketopiperazines (ETPs) are a broad class of fungal metabolites that display potent antibiotic and cytostatic/cytotoxic activities [19]. Various molecular mechanisms have been proposed explaining the biological activity of ETPs, e.g. (I) enzyme inhibition through ETP conjugation with cysteine residues, (II) ejection of a structurally important zinc ion from a transcription factor, (III) increased levels of cellular reactive oxygen species through competitive inhibition of thioredoxin reductase, (IV) inhibition of heat shock protein 90 through induction of a conformational change [20–23]. To date, chaetocin is by far the most widely investigated ETP with anticancer activity in solid tumors and hematological malignancies [24–26]. Chaetocin was the first reported specific S-Adenosyl-methionine (SAM)-competitive inhibitor of the histone methyltransferase SUV39H1 [27], gaining much attention since deregulated epigenetic modifiers such as histone methyltransferases (HMTs) and DNA methyltransferases (DNMTs) have recently emerged as promising new drug targets [28]. SUV39H1 inhibition with chaetocin or RNAi-mediated SUV39H1 knockdown led to reduced tri-methylation of lysine 9 in histone 3 (H3K9me3), re-expression of silenced tumor suppressor genes and apoptosis induction *in vitro* and reduced tumor growth *in vivo* [26, 29]. Recently chaetocin has also been shown to not only reduce H3K9me3 levels, but also tri-methylation of lysine 27 in histone 3 (H3K27me3) in AML cell lines [30]. Tri-methylation of H3K27 is catalyzed by EZH2, the HMT in the polycomb repressive complex 2 (PRC2), which also controls DNA methylation through its interaction with DNMTs [31]. Deregulation of both EZH2 and DNMTs has been reported in AML, resulting in histone and DNA hypermethylation and consequently aberrant silencing of tumor suppressor genes such as *BIM* and *CDKN2B (p15)* [32, 33].

Here, we report that a novel ETP, NT1721, possesses potent antileukemic activity, which is mediated by the depletion of epigenetic modifiers (DNMT1, EZH2, BMI1). NT1721 showed potent antileukemic effects as single agent and in combination with drugs currently used for AML treatment. Moreover, NT1721 was highly efficacious and well-tolerated in a systemic AML mouse model, highlighting its potential as novel agent for the treatment of AML.

## RESULTS

### NT1721 potently reduced the cell viability of AML cell lines and primary AML samples

**Figure 1:**
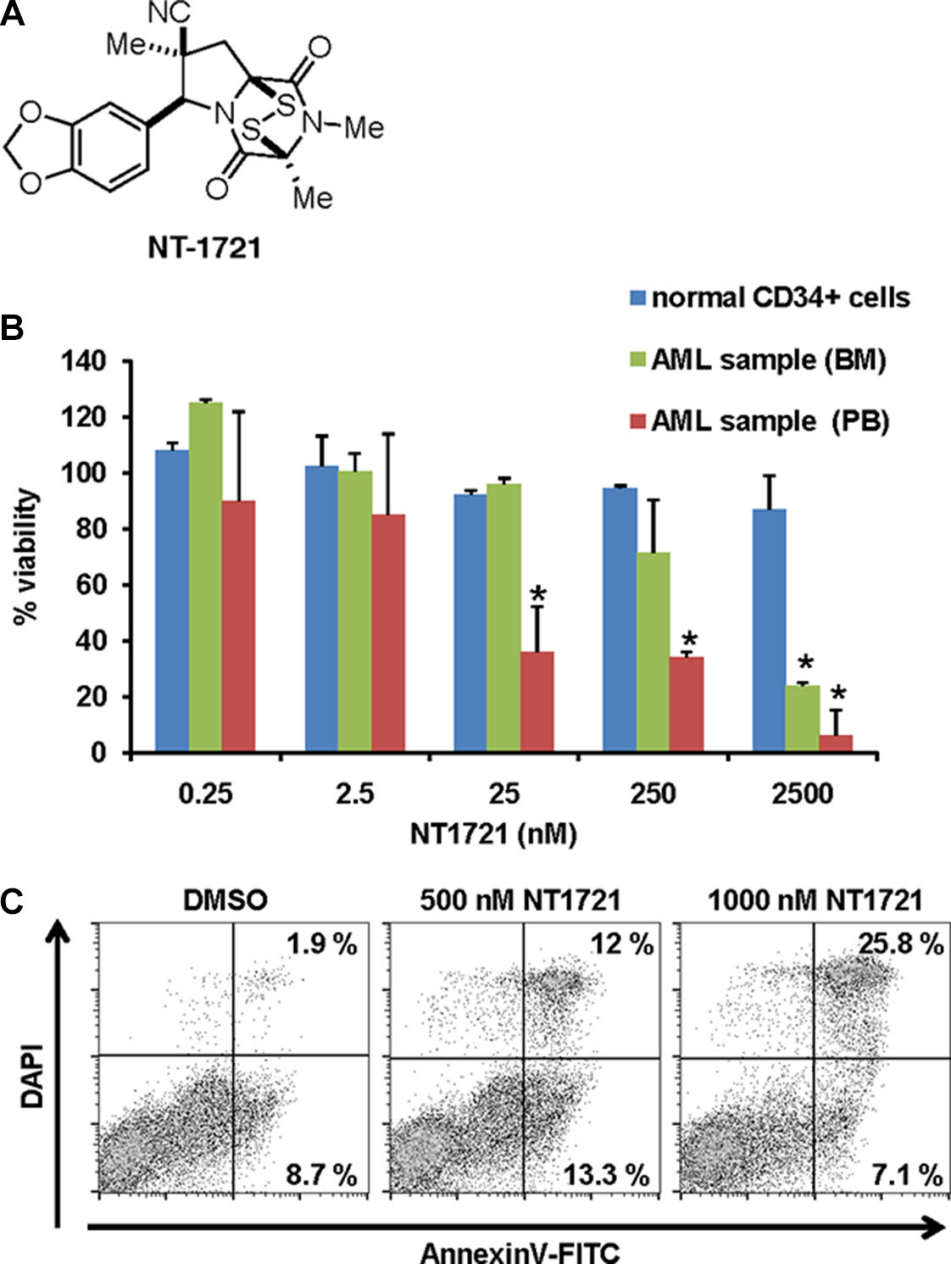
Effect of NT1721 on the viability of primary AML samples and normal CD34+ bone marrow cells (**A**) Chemical structure of NT1721. (**B**) The cell viability of normal CD34^+^ bone marrow cells and primary AML samples from peripheral blood (PB) or bone marrow (BM) of two newly diagnosed AML patients was determined after 48 h of treatment with NT1721. The graphs represent the mean ± SD from three experiments. The asterisks indicate statistical significant differences compared to the untreated control (*P* values < 0.05). (**C**) Apoptosis induction. Primary AML cells from bone marrow were treated with NT1721 for 48 h and stained with annexin V.

UCI1406 was chosen for this study from a library of ETPs because of its potent antitumor activity in various tumor cell lines [34]. To determine the potency of the racemic mixture (UCI1406) and its enantiomers (NT1721 and NT1722, Figure 1A) against AML we determined their IC_50_ values in Molm14 cells that were treated with the respective compounds for 48 h. Cells treated with NT1721 displayed a 9-fold lower IC_50_ value than cells treated with NT1722 (Table 1), indicating that NT1721 had better anti-leukemic properties than NT1722. Thus, NT1721 was used for all further experiments. To assess the effect of NT1721 on the viability of various AML cell lines, we treated FLT3-ITD cells (Molm14, MV4.11) and FLT3-WT cells (THP1, KG1a) with increasing concentrations of NT1721 (0.1 nM - 10 μM) and determined the IC_50_ values after 48 h. Molm14, MV4.11 and THP1 cells displayed IC_50_ values in the lower nanomolar range (Table 1). By contrast, the IC_50_ value was significantly higher (∼ 8 μM) in KG1a leukemic stem-like cells, which reportedly possess many characteristics of leukemic stem cells, e.g. self-renewal capacity, resistance to chemotherapy and a CD34^+^/CD38^−^ phenotype [35, 36]. Moreover, CD96 has also been identified as a leukemic stem cell-specific marker on AML CD34^+^/CD38^−^ stem cells [37]. To confirm that KG1a cells display the immunophenotype associated with AML stem-like cells, we assessed the expression of CD34, CD38 and CD96 in KG1a cells; FACS analysis showed that 74% of KG1a cells were CD34^+^/CD38^−^/CD96^+^ (Supplementary Figure S1A). CD96 was strongly expressed in the majority of KG1a cells while only weak CD96 expression was detected ∼2% of CD38^−^ Molm14 or normal CD34^+^ bone marrow cells. Taken together these data suggest that KG1a cells may be enriched for leukemic stem-like cells. We then compared the effect of NT1721 on the cell viability with that of drugs currently used for AML treatment, i.e. the standard remission induction drug, cytarabine, and sorafenib, a TKI that has been used in clinical trials in patients harboring FLT3-ITD mutations [38]. The IC_50_ values for cytarabine and sorafenib were 6 to 500-fold higher (depending on the cell line) than the IC_50_ values for NT1721 in both FLT3-ITD and FLT3-WT cell lines (Table 1). KG1a cells showed no response to 48 h-treatment with either 10 μM cytarabine or 10 μM sorafenib. When we extended the exposure time to 96 h, KG1a cells displayed an IC_50_ value of ∼1 μM for NT1721, but still showed no significant change in viability after treatment with cytarabine or sorafenib (Table 1). The result was confirmed when we stained treated KG1a cells with annexin V to detect apoptotic cells; 40% of KG1a cells treated with 1 μM NT1721 were annexin V^+^ compared to 5–8% of cells treated with 10 μM sorafenib or 10 μM cytarabine (Supplementary Figure S1B). Taken together, these data indicate that NT1721 is more potent than either cytarabine or sorafenib *in vitro*.

**Table 1:** IC_50_ values in AML cell lines

IC_50_ (nM)					
	UCI1406	NT1722	NT1721	sorafenib	cytarabine
**Molm14**	3.4 ± 1.0	22.4 ± 6.7	2.5 ± 0.6	214 ± 68	1228 ± 384
**MV4.11**	n/d	n/d	6.6 ± 1.3	40 ± 6	935 ± 107
**THP1**	n/d	n/d	169 ± 70	7939 ± 1319	1148 ± 329
**KG1a**	n/d	n/d	8310 ± 346	> 10 μM	> 10 μM
**KG1a (96 h)**	n/d	n/d	983 ± 152	> 10 μM	> 10 μM

AML cell lines were treated with various drug concentrations (up to 10 μM); the cell viability and IC_50_ values were determined after 48 h and 96 h (KG1a only). The IC_50_ values represent the mean ± SD from at least three independent experiments. The *P* values for comparing the IC_50_ values for NT1722 with NT1721 or UCI1406 were < 0.01.

We then tested the effect of NT1721 on primary AML samples (from AllCells, obtained from two newly diagnosed AML patients with white blood cell counts > 10^11^/L) by treating the samples with NT1721 for 48 h. The IC_50_ values for the primary AML peripheral blood and AML bone marrow sample (∼10 nM and ∼1 μM, respectively) were comparable to the IC_50_ values achieved in AML cell lines (Figure 1B). Moreover, NT1721 induced apoptosis as measured by the dose-dependent increase in annexin V-positive cells to 33% in the AML bone marrow sample treated with 1 μM NT1721 (Figure 1C). To assess the effect of NT1721 on normal cells we also treated normal CD34^+^ bone marrow cells with NT1721 for 48 h. This treatment had only a comparatively minor effect on their viability since 84% of the normal CD34^+^ bone marrow cells remained viable after exposure to 2.5 μM NT1721 for 48 h (Figure 1). Taken together, our results with primary cells and AML cell lines suggest that NT1721 may preferentially decrease the viability of AML cells while leaving normal CD34^+^ bone marrow largely unaffected.

### NT1721 enhances the cytotoxic effects of cytarabine or sorafenib in FLT3-ITD AML cells

**Figure 2:**
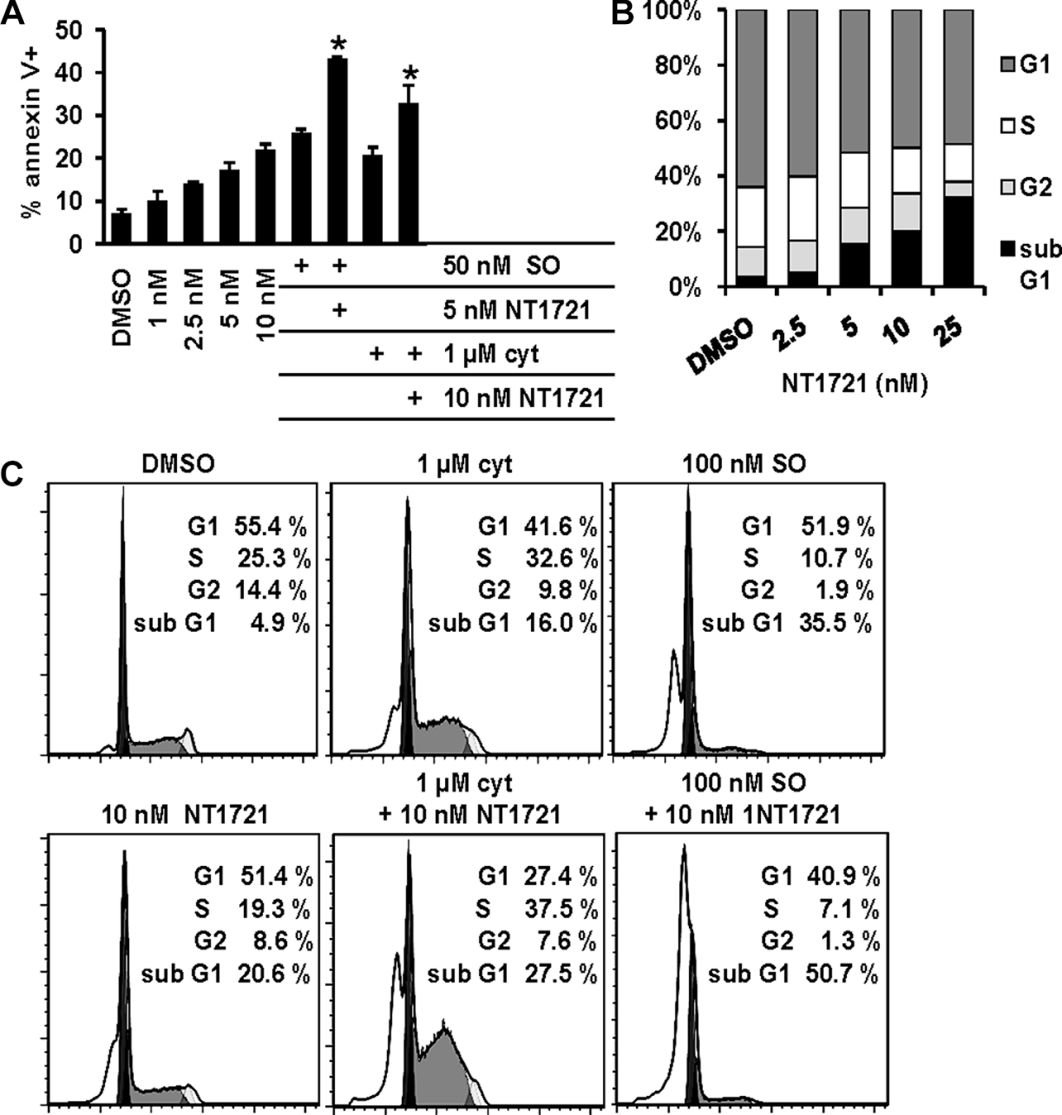
Drug combinations of NT1721 with cytarabine or sorafenib display better antileukemic properties than the single agents (**A**) Apoptosis induction. Molm14 cells were treated for 48 h with the single agents or the drug combinations as indicated, stained with annexin V and analyzed by flow cytometry. The graphs represent the mean ± SD from three independent experiments. The asterisks indicate statistical significant differences compared to the respective single agents (*P* values < 0.01). (**B** and **C**) Cell cycle analysis. Molm14 cells were treated for 48 h, fixed, stained with PI and subjected to flow cytometry; the data were analyzed with FlowJo software using the Watson model.

To evaluate the cytotoxic effect of drug combinations of NT1721 with either cytarabine or sorafenib, we treated Molm14 cells with increasing concentrations of the single agents or drug combinations (NT1721-to-cytarabine ratio 1:100 and NT1721-to-sorafenib ratio 1:10) for 48 h. Calculation of the combination index (CI) revealed synergistic effects (CI < 1) for the combinations with cytarabine or sorafenib at the effective doses ED50, ED75 and ED90 (Table 2 and Supplementary Figure S2). At ED25, the combination with cytarabine displayed a CI < 1 while no synergy was observed for the combination with sorafenib. To further investigate the antileukemic effects of NT1721 and the drug combinations, we determined the effect of the single agents and drug combinations on apoptosis induction and cell cycle after 48 h exposure. As shown in Figure 2A, treatment with NT1721 increased the percentage of annexin V^+^, apoptotic cells in a dose-dependent manner. Cells treated with drug combinations of NT1721 with cytarabine or sorafenib displayed higher percentages of apoptotic cells than cells treated with the single agents, suggesting that NT1721 may augment their antileukemic effect. The cell cycle analysis revealed that NT1721 increased the sub-G1 fraction in a dose-dependent manner and decreased the percentage of cells in the G1-, S- and G2 phase (Figure 2B), indicating that NT1721 did not only lead to cell cycle arrest, but induced cell death. As shown in Figure 2C, cells treated with drug combinations displayed higher percentages of sub-G1 phase cells than the single agents, suggesting that NT1721 enhanced the effect of cytarabine and sorafenib.

**Table 2:** Calculation of the combination index (CI)

Combination index (CI)	ED25	ED50	ED75	ED90
**NT1721:cyt (ratio 1:100)**	0.321	0.27	0.303	0.389
**NT1721:SO (ratio 1:10)**	1.075	0.318	0.223	0.189

Molm14 cells were treated with the single agents or drug combinations for 48 h. Data from cell viability assays were used to determine the CI for the drug combinations at the effective doses ED25, ED50, ED75 and ED90. CI values < 1 indicate synergy.

### High concentrations of NT1721 decreased H3K9me3 levels

Since NT1721 is structurally related to the known SUV39H1 inhibitor chaetocin, we tested first whether treatment with NT1721 altered the methylation status of H3K9 in Molm14 cells. As shown in Supplementary Figure S3, NT1721 only slightly decreased global H3K9me3 levels (by ∼20%) at concentrations 10-fold higher than the IC_50_ value for Molm14. NT1721 also displayed a relatively high IC_50_ value of 1.3 μM when we tested its ability to inhibit the HMT activity of recombinant SUV39H1 in a cell-free assay (data not shown). These results suggest that the antileukemic effect of NT1721 is not primarily mediated by SUV39H1 inhibition.

### NT1721 depleted EZH2, DNMT1, and BMI1 protein levels and induced the expression of tumor suppressor genes

**Figure 3:**
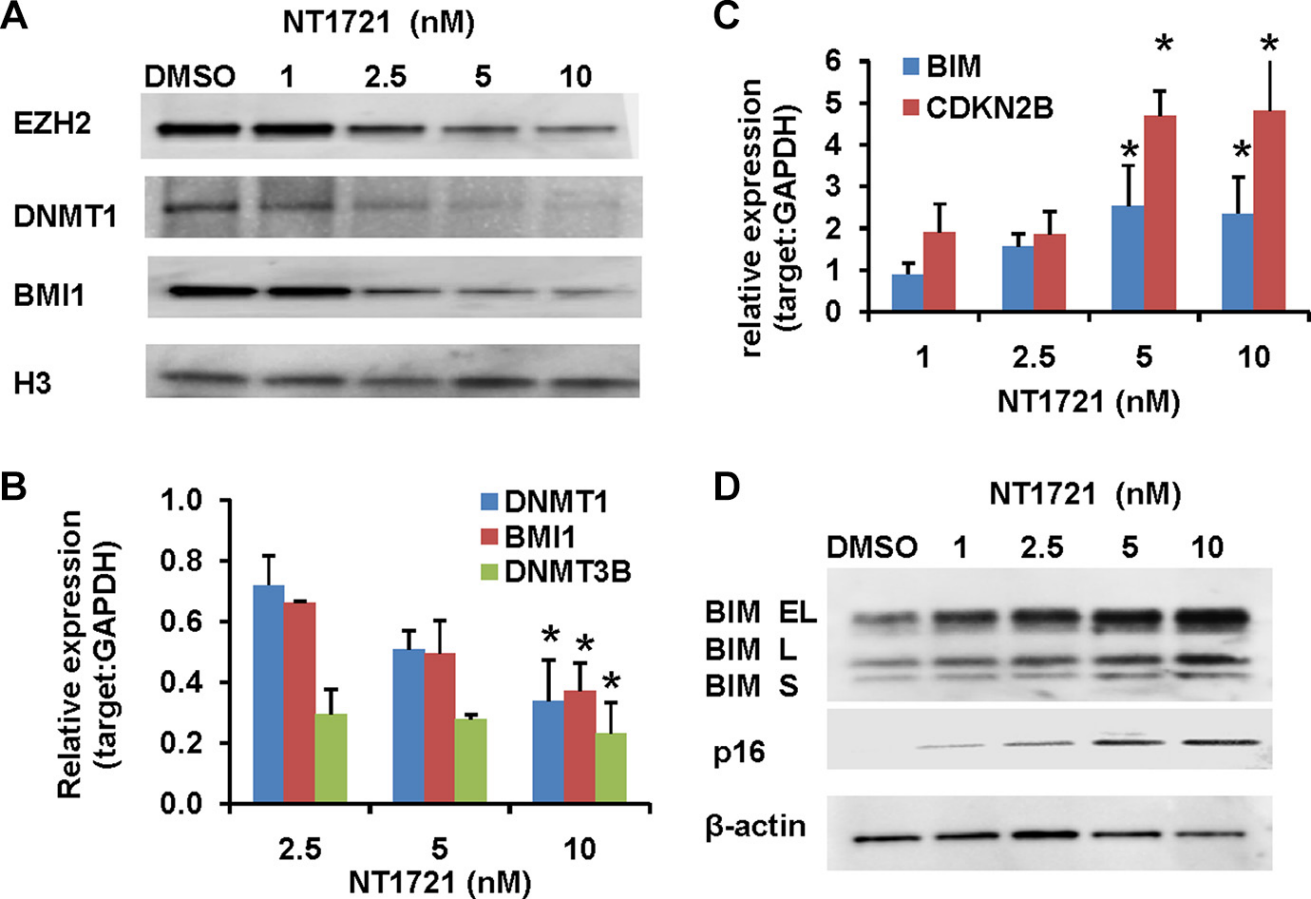
Treatment with NT1721 led to the depletion of DNMT1, EZH2 and BMI1 and induced the expression of tumor suppressor genes Molm14 were treated with NT1721 as indicated for 24 h. (**A**) Western blot analysis of EZH2, DNMT1, and BMI1 expression. (**B**) QPCR analysis of DNMT1, DNMT3B and BMI1 expression. The data were analyzed using GAPDH as reference gene. The graphs represent the mean ± SD from at least three independent experiments. The asterisks indicate statistical significant differences compared to the untreated controls (*P* values < 0.05). (**C**) QPCR analysis of the expression of tumor suppressor genes, *CDKN2B (p16)* and *BIM*. The graphs represent the mean ± SD from three independent experiments. The asterisks indicate statistical significant differences compared to the untreated control (*P* values < 0.05). (**D**) Western blot analysis of CDKN2A (p16) and BIM expression.

Since chaetocin has been recently shown to reduce global H3K27me3 levels in AML cell lines [30], we investigated the effect of NT1721 on H3K27 methylation in Molm14 cells. Western blot analysis revealed that NT1721 led to a greater reduction in H3K27me3 levels than in H3K9me3 levels at low concentrations (Supplementary Figure S3). However, NT1721 had no direct inhibitory effect on the HMT activity of recombinant EZH2, the HMT catalyzing tri-methylation of H3K27 as measured in a cell-free assay (data not shown). We then investigated whether NT1721 influenced the expression level of EZH2. As shown in Figure 3A, treating Molm14 cells with low nanomolar concentrations of NT1721 led to a significant, concentration-dependent decrease in EZH2 protein levels, thus providing an explanation for the decrease in H3K27me3 levels.

To gain further insight in how NT1721 might exert its antileukemic activity we analyzed RNAseq data obtained from cells treated with NT1721; the analysis showed that NT1721 decreased the expression of several epigenetic regulators (i.e. *DNMT1, DNMT3B* and *BMI1*) that are linked to the silencing of tumor suppressors and the maintenance and self-renewal of leukemic stem cells [39]. We first verified the downregulation of *DNMT1, DNMT3B* and *BMI1* in Molm14 cells treated with NT1721 by qPCR (Figure 3B). Western blot analysis then confirmed that treatment with NT1721 depleted DNMT1 and BMI1 protein levels in a dose-dependent manner (Figure 3A). Treatment with NT1721 at IC_50_ concentrations also greatly reduced DNMT1 (by > 60%) and BMI1 expression (by 70–85%) in the primary AML samples. We then investigated whether NT1721 influenced the expression of tumor suppressor genes since several reports have shown links between the downregulation of DNMT1, BMI1 and EZH2 and increased expression of tumor suppressor genes [40–42]. Western blot and qPCR analysis showed that depletion of DNMT1, EZH2 and BMI1 was associated with a concomitant increase in the expression of tumor suppressor genes, i.e. CDKN2B (p15), BIM and CDKN2A (p16) (Figure 3C–3D). NT1721 also decreased the expression of the antiapoptotic protein BCL2 and the cyclin-dependent kinase 2 (CDK2), which is essential for entry into the cell cycle and thus for cell proliferation, in a concentration-dependent manner (data not shown). This result is in agreement with previous reports showing that BMI1 knockdown decreased expression of CDK2 and BCL2 and reduced cell proliferation of various types of cancer [43–45].

### NT1721 decreased FLT3LG expression and attenuated cytarabine-induced FLT3LG upregulation

**Figure 4:**
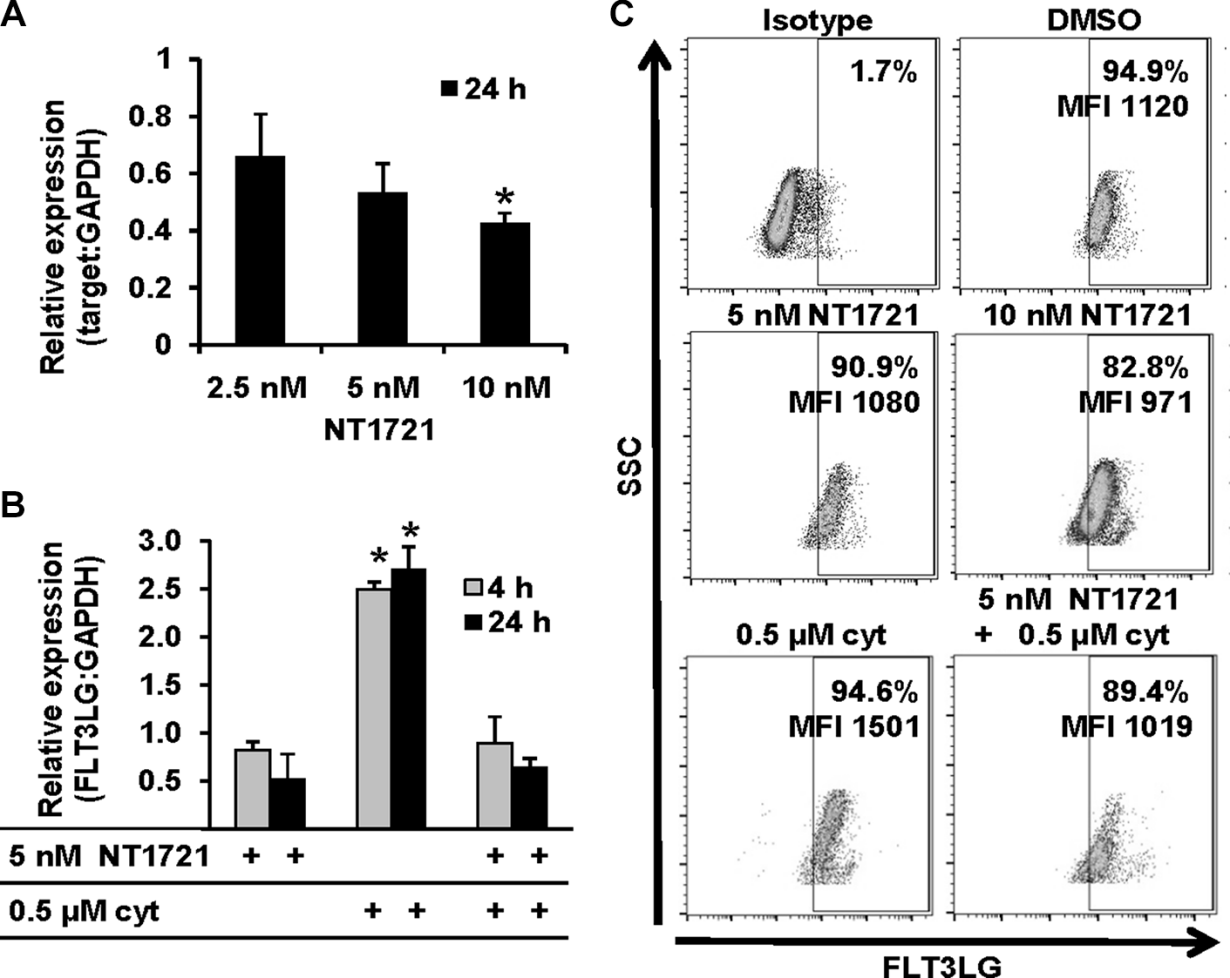
NT1721 attenuated the cytarabine-induced FLT3LG upregulation Molm14 were treated with NT1721 and cytarabine as indicated. (**A** and **B**) qPCR analysis of FLT3LG expression.The data were analyzed using GAPDH as reference gene. The graphs represent the mean ± SD from three independent experiments. The asterisks indicate statistical significant differences compared to the untreated controls (*P* values < 0.01). (**C**) FACS analysis of FLT3LG expression in Molm14 cells.

Analysis of the RNAseq data also revealed that FLT3 ligand (*FLT3LG*) expression was decreased in treated cells, which was confirmed by qPCR analysis in Molm14 cells (Figure 4A). Treatment with NT1721 at IC_50_ concentrations also reduced the *FLT3LG* expression by ∼70% in the primary AML samples. Since the chemotherapy-induced FLT3LG surge is linked to drug resistance to TKIs [14, 16], we tested whether NT1721 could influence the cytarabine-induced FLT3LG upregulation. As shown in Figure 4B, treatment with cytarabine increased *FLT3LG* mRNA levels and co-treatment with NT1721 attenuated the cytarabine-induced upregulation of *FLT3LG* in Molm14 cells. To confirm this result on the protein level we analyzed the FLT3LG expression by flow cytometry. NT1721 decreased the FLT3LG expression in a dose-dependent manner in FLT3-ITD^+^ Molm14 cells (Figure 4C) and FLT3-WT KG1a cells (Supplementary Figure S4), as evidenced by the decrease in FLT3LG^+^ cells and in the median fluorescence intensity (MFI). Co-treatment with NT1721 attenuated the cytarabine-induced increase in FLT3 expression (Figure 4C).

### NT1721 showed potent antileukemic effects in a systemic FLT3-ITD mouse model

We first determined the maximum tolerated dose (MTD) of NT1721 in NSG mice by administering 7.5 mg/kg, 15 mg/kg or 30 mg/kg of NT1721 by gavage. Daily doses up to 15 mg/kg were apparently well tolerated since no loss of body weight or other obvious signs of toxicity were observed. Mice receiving daily doses of 30 mg/kg of NT1721 displayed significant weight loss (> 15%) and diarrhea after 4 to 5 days of daily treatment. However, no significant weight loss was observed when the mice received 30 mg/kg of NT1721 on only 3 consecutive days per week, suggesting that this drug regimen was well tolerated (Supplementary Figure S5).

**Figure 5:**
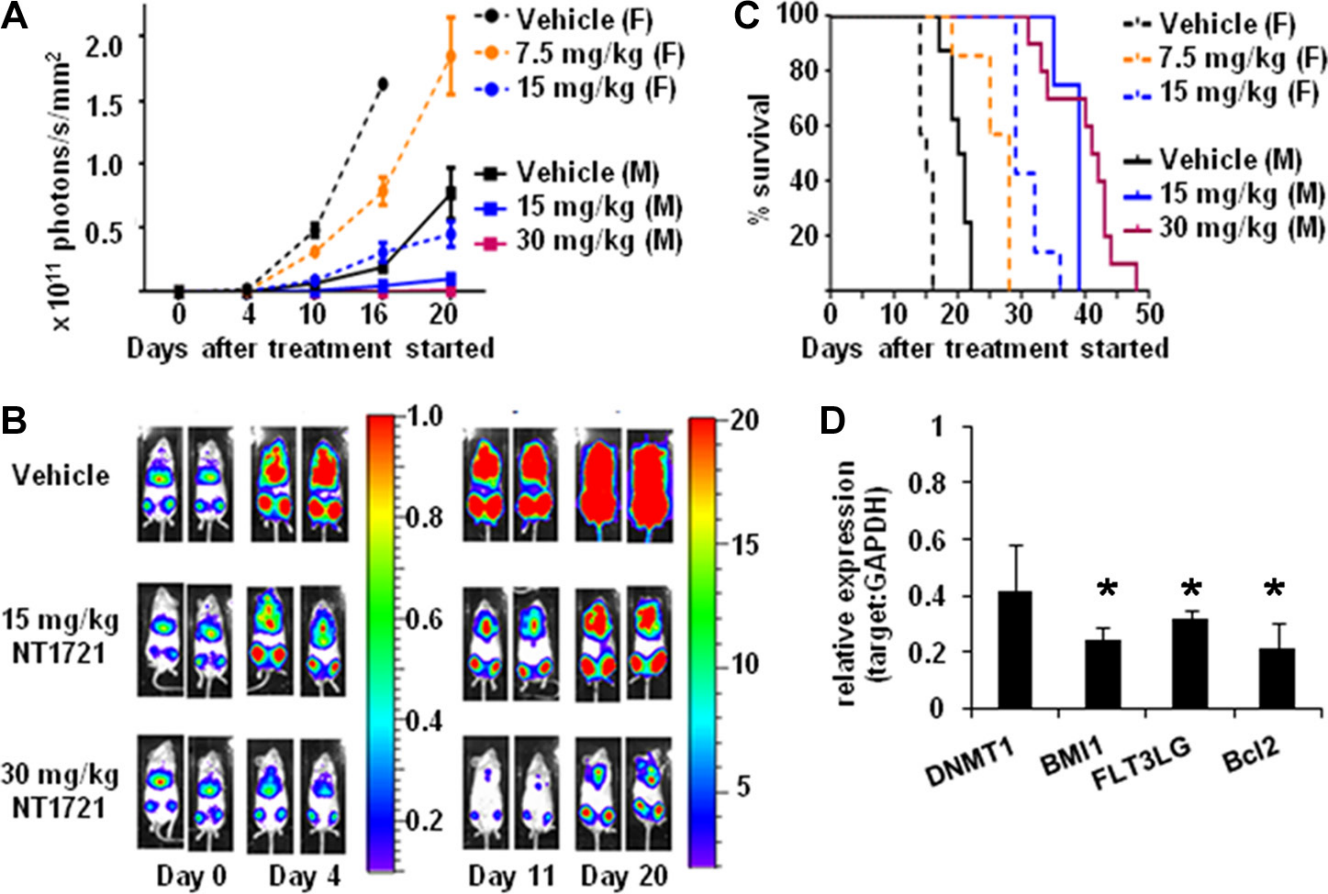
Efficacy of NT1721 in a systemic AML mouse model NSG mice were injected (I.V.) with 10^6^ luc^+^ Molm14 cells. Four days later mice were divided into groups bearing equal tumor burdens and treated by gavage with the indicated doses of NT1721 or the vehicle control. (**A**) *In vivo* monitoring of bioluminescent signals. Female (F) and male (M) mice were treated as indicated. Bioluminescent signals were monitored on the indicated days and expressed as photons/s/mm^2^ ± SD. (**B**) Bioluminescent signals in male NSG mice. Two representative mice from each treatment group are shown. The color bars show the relative luciferase activity in 10^6^ photons/s/mm^2^. (**C**) Survival curves. Groups of female and male NSG mice (at least 3 per group) were treated with the indicated doses of NT1721 or the vehicle control until they died or met the criteria for euthanasia. (**D**) Changes in gene expression *in vivo*. Human cells were isolated from the bone marrow of individual mice after 14 days of treatment (daily dose of 15 mg/kg of NT1721 or vehicle, *n* = 3/group). Purified total RNA from individual mice was used for qPCR assays. The graphs represent the mean ± SD. The asterisks indicate statistical significant differences compared to the control group (*P* values < 0.01).

To evaluate the antileukemic activity of daily doses of NT1721 *in vivo*, we injected 10^6^ luciferase-expressing (luc^+^) Molm14 cells into the tail vein of female NSG mice. Groups of mice bearing equal tumor burdens after 4 days were then treated daily with 7.5 mg/kg or 15 mg/kg of NT1721 by gavage; the control group received the vehicle control (30% Solutol/5% DMSO in PBS). As shown in Figure 5A and 5B, treatment with NT1721 led to dose-dependent lower tumor burdens in treated mice compared to the control. The tumor burden in mice treated with 15 mg/kg of NT1721 was 70–80% lower than the tumor burden in the control group (days 10 and 16). Treatment with NT1721 significantly improved the survival time; control mice died after 14 days, while treatment with 15 mg/kg of NT1721 doubled the median survival time to 29 days (Figure 5C). We then compared the efficacy of daily treatment with NT1721 (15 mg/kg) to treatment with 30 mg/kg of NT1721 administered on 3 consecutive days per week in NSG mice (male and female, injected with 10^6^ luc^+^ Molm14 cells). Tumors grew slower in male compared to female mice (Figure 5A). However, daily doses of 15 mg/kg of NT1721 suppressed tumor growth by 70–80% in both male and female mice compared to the respective control mice (day 16 and 20, Figure 5A). Treatment on 3 consecutive days with 30 mg/kg of NT1721 was significantly more efficacious compared to daily doses of 15 mg/kg; mice receiving 30 mg/kg of NT1721 displayed a > 95% lower tumor burden compared to the control (days 16 and 20, Figure 5A). Median survival times also increased from 39 days for mice receiving 15 mg/kg to 42 days for mice receiving 30 mg/kg (Figure 5C). Taken together, these data indicate that NT1721 potently inhibited AML proliferation *in vivo* and significantly increased survival times.

To investigate the effect of NT1721 on gene expression levels *in vivo*, we sacrificed mice from the control and treatment groups (daily doses of 15 mg/kg) after 14 days. We then isolated human CD45^+^ cells from the bone marrow of individual mice (*n* = 3/group) and assessed the changes in gene expression by qPCR. As shown in Figure 5D, treatment with NT1721 significantly decreased the expression of *DNMT1, BMI1, FLT3LG* and *BCL2* in treated mice compared to the control, recapitulating the changes we observed *in vitro*.

## DISCUSSION

Clinical outcome for AML patients, especially for those harboring FLT3-ITD mutations, remains poor despite initial responses to chemotherapy with cytarabine and treatment with FLT3 inhibitors [12, 38]. Increasing drug resistance over the course of the treatment and the persistence of leukemic stem cells are thought to be major causes for high relapse and poor survival rates, highlighting the need for new treatment options [46].

Aberrant DNA and histone methylation patterns are common in AML patients and can be used to predict treatment outcome, thus underscoring the important role of epigenetic mechanisms in AML development [47]. Previous reports show that significantly increased BMI1 expression in CD34^+^ cells in the peripheral blood and bone marrow of AML patients compared to normal cells is associated with worse prognosis [39, 48, 49]. DNMT1, EZH2 and BMI1 inhibition or knockdown induced re-expression of silenced tumor suppressor genes and apoptosis and importantly, repression of BMI1 decreased self-renewal and maintenance of leukemic stem cells [32, 39, 42, 50]. Hence, epigenetic modifiers have increasingly been recognized as promising drug targets in AML. Here, we show that treatment of AML cells with a novel ETP, NT1721, depleted DNMT1, EZH2 and BMI1 and concomitantly increased the expression of the tumor suppressor genes CDKN2A (p16), CDKN2B (p15) and BIM: Up-regulation of BIM may enhance apoptosis induction since a previous study showed that silencing of BIM promotes apoptosis resistance in leukemia [51]. In AML, inactivation has been reported for both *CDKN2A (p16)* and most frequently *CDKN2B (p15)* [33, 52]. While BMI1 depletion is associated with the re-expression of CDKN2A (p16) [39, 53], DNMT1 depletion might partially explain the increased expression of *CDKN2B (p15)*: Previous reports showed a correlation between *CDKN2B (p15)* methylation and higher DNMT1 expression as well as an association of DNMT1 downregulation and p15 re-expression in AML [33, 42]. Moreover, depletion of the epigenetic modifiers concomitantly increased the expression level of several miRNAs (i.e. *miR148a, miR214* and *miR200c*) that target *DNMT1, EZH2* and *BMI1* [41, 54] (data not shown). The increased miR expression could partially explain the downregulation of the epigenetic modifiers since a previous study demonstrated that DNMT1 depletion following treatment with the DNMT1 inhibitor, 5-azacitidine, increased the expression of miR214 and miR200c in cord blood-derived multipotent stem cells [41]. However, NT1721 does not directly inhibit DNMT1 as it has no inhibitory effect on DNMT1 in a cell-free assay (data not shown). Furthermore, it seems unlikely that the antileukemic effect of NT1721 is mainly mediated by SUV39H1 inhibition given the big difference in IC_50_ values between cell-based viability assays (lower nanomolar range for Molm14, MV4.11 and THP-1) and the SUV39H1 inhibition in a cell-free assay (1.3 μM). Thus, further studies are needed to understand the mechanism of action and determine the direct molecular target of NT1721.

Treatment of AML cells with NT1721 was more efficacious than treatment with cytarabine and sorafenib, regardless of the FLT3 mutation status. Importantly, NT1721 also induced apoptosis in CD34^+^/CD38^−^/CD96^+^ KG1a leukemic stem-like cells that displayed resistance to clinically used AML drugs, i.e. cytarabine and sorafenib, suggesting that NT1721 may be a valuable new agent for the treatment of drug-resistant AML. Recent studies show that combining cytarabine with drugs that target epigenetic modifiers (i.e. DNMT1 and HDAC inhibitors) may be a promising new strategy for AML treatment [55, 56]. Here we show that combinations of NT1721 with cytarabine induced apoptosis and cell death more effectively than the single agents. Moreover, targeting FLT3 alone may not be sufficient to successfully treat AML since FLT3-ITD remains responsive to FLT3LG [8, 12, 13]. Autoactivation of FLT3-ITD in absence of FLT3LG is weak, which suggests that FLT3-ITD is hyperresponsive to its ligand rather than autoactivated [16]. Thus, the chemotherapy-induced FLT3LG surge is thought to be a possible cause for transient responses to treatment with single FLT3 inhibitors. Here we show that NT1721 attenuated the cytarabine-induced FLT3LG up-regulation and that combining the FLT3 inhibitor sorafenib with NT1721 induced apoptosis and cell death to a greater degree than the single agents. Our results indicate that targeting both FLT3 and FLT3LG may be more effective than FLT3 inhibition alone.

Taken together, our results suggest that NT1721 may be a valuable new agent for the treatment of AML regardless of the FLT3 mutation status. NT1721 (used either as a single agent or in combination with chemotherapy or TKIs) could potentially improve the response of drug-resistant AML blasts and leukemic stem cells through the induction of silenced tumor suppression genes and downregulation of genes promoting self-renewal of leukemic stem cells. Our *in vitro* results and the potent *in vivo* activity of NT1721 in a systemic AML model provide a rational for future studies to test the clinical efficacy of NT1721 against AML.

## MATERIALS AND METHODS

### Reagents

The ETPs (UCI1406, NT1721, NT1722) were synthesized as previously described (Figure 1A) [34].

### Cell culture

All cell lines were obtained from ATCC or the Leibniz Institute DSMZ (German Collection of Microorganisms and Cell Cultures), authenticated by STR-profiling at the sources and passaged for less than 6 months after receipt or resuscitation. MV4.11 and KG1a cells were maintained in IMDM medium supplemented with 10% or 20% FBS (omega scientific), respectively; THP1 and Molm14 cells were maintained in RPMI medium/10% FBS. Normal CD34^+^ bone marrow cells and primary AML samples from patients' peripheral blood or bone marrow were obtained from AllCells. All primary cells were cultured in IMDM/10% FBS.

### Determination of IC_50_ values and synergism

20,000 cells/well were seeded in 96-well plates and treated with a wide range of concentrations (0.1 nM - 10 μM) of NT1721, cytarabine (R&D Systems), sorafenib (LC laboratories) or drug combinations as indicated. After 48 h, the cell viability was determined using the MTS assay (CellTiter 96^®^ AQueous One Solution, Promega) according to the manufacturer's instructions. Data from the MTS assay were expressed as percent of viable cells compared to the vehicle control (0.3% DMSO). IC_50_ values were calculated from the dose response curve using GraphPad Prism 6 software. To quantify synergistic, additive or antagonistic effects of the drug combinations, the combination indexes (CI) were calculated using Calcusyn software (Biosoft), where CI < 1, CI = 1, and CI > 1 indicate synergistic, additive, and antagonistic effects, respectively.

### Apoptosis assay and cell cycle analysis

Cells were treated with the drugs or drug combinations and harvested after 48 h. For the apoptosis assay, cells were stained with annexin V (BD Biosciences) according to the manufacturer's instructions. For the cell cycle analysis, cells were fixed in 70% ethanol for 1 h at 4°C, washed twice with PBS, treated with RNAse A at 37°C for 1 h and stained with propidium iodide (BD Biosciences). Fluorescence data were collected on a CyAN flow cytometer (Beckman Coulter) and analyzed with FlowJo software (TreeStar). The cell-cycle distribution was determined using the Watson (pragmatic) fit option in FlowJo.

### Flow cytometry for cell surface markers

To determine the expression of CD34, CD38 and CD96 cells were stained with the following antibodies according to the manufacturer's instructions: PE-CD34, FITC-CD38 (BD Biosciences), PE-CD96 (Miltenyi, San Diego, CA) or an isotype control. Fluorescence data were analyzed with FlowJo software.

### QPCR

Total RNA was isolated using the Direct-zol (Zymo Research) kit and reverse transcribed using the Tetro cDNA synthesis kit (Bioline). The following qPCR primers were used:

CDKN2B: 5′gcggggactagtggagaag/5′ctgcccatcatcatgacct; BCL2: 5′agtacctgaaccggcacct/5′gccgtacagttccacaaagg; BIM: 5′catcgcggtattcggttc/5′gctttgccatttggtcttttt; BMI1: 5′ccattgaattctttgaccagaa/5′ctgctgggcatcgtaagtatc; DNMT1: 5′caaacccctttccaaacctc/5′taatcctggggctaggtgaa; DNMT3B:5′agagggacatctcacggttc/5′ggttgccccagaagtatcg; FLT3LG: 5′ctggatcactcgccagaact/5′tggcagggttgaggagtc; GAPDH: 5′agccacatcgctcagacac/5′gcccaatacgaccaaatcc

Quantitative PCR was performed using a CFX96 Touch Real-Time PCR detection system (Bio-Rad). Relative expression levels were calculated using the 2^−ΔΔCt^ method and GAPDH as reference gene.

### Western blots

Cell lysates were prepared using RIPA buffer (Sigma)/Halt protease inhibitor cocktail (Thermo Fisher, Rockford, IL) and the protein concentrations were determined using the BCA protein assay (Thermo Fisher). Equal protein amounts were subjected to SDS-PAGE and transferred onto PVDF membranes (Bio-Rad). Immunodetection was performed using the following primary antibodies: BIM, BMI1, DNMT1, EZH2, Histone3, β-actin (Cell signaling), H3K9me3, H3K27me3, CDKN2A (p16) (abcam) and the appropriate ECL HPR-linked secondary antibodies (GE healthcare).

### Lentiviral vectors

To generate a luciferase-expressing lentiviral plasmid, the plasmid pGL4.10[*luc2*] (Promega) was used as PCR template to amplify the luciferase (*luc2)* gene. The PCR product was cloned into the lentiviral plasmid pLVX-EF1α-IRES-puro (Clontech). Luciferase-expressing lentiviral vectors were then generated by calcium phosphate co-precipitation. Molm14 cells were transduced with the pLV-luc2-puro vectors at an MOI of 0.5 and selected with 1 μg/ml puromycin (Sigma).

### *In vivo* studies

Mouse care and experimental procedures were performed under pathogen-free conditions in accordance with approved protocols from the institutional animal care and use committee of City of Hope National Medical Center. For the systemic AML model, 10^6^ luciferase-expressing Molm14 cells were injected into the tail vein of 6-to 8-week old NSG mice (Jackson Laboratory). To determine the leukemic burden, we injected the mice (I.P.) with 3 mg D-Luciferin (Promega) 4 days after tumor injection and imaged them in an IVIS 100 (Caliper Life Sciences). A standard region of interest (ROI), which included the entire mouse, was used to determine the total body bioluminescence. Data were expressed as photons/s/mm^2^. The mice were then distributed into groups bearing equal tumor burdens and treated by gavage with NT1721 or the vehicle control (5% DMSO/30% solutol (Sigma)) as indicated.

To assess changes in gene expression levels in the human cells, we sacrificed mice after 14 days of treatment, extracted the bone marrow cells and isolated human cells using anti-human CD45 microbeads (Miltenyi) according to the manufacturer's protocol. The RNA was then isolated and used for qPCR assays.

### Statistical analysis

The 2-tailed Student's *t*-test was used to determine statistical significance between two treatment groups; *P* values < 0.05 were considered to be significant. The log-rank test was used to evaluate the statistical significance in survival curves.

## SUPPLEMENTARY MATERIALS FIGURES


