# Supplementary material for: NT1721, a novel epidithiodiketopiperazine, exhibits potent in vitro and in vivo efficacy against acute myeloid leukemia
Source: Oncotarget. 2016 Nov 15;7(52):86186–97. doi: 10.18632/oncotarget.13364 (PMC5349906; doi:10.18632/oncotarget.13364)
Supplement: Supplementary file 1 [file oncotarget-07-86186-s001.pdf]

NT1721, a novel epidithiodiketopiperazine, exhibits potent *in vitro* and *in vivo* efficacy against acute myeloid leukemia

Supplementary Materials

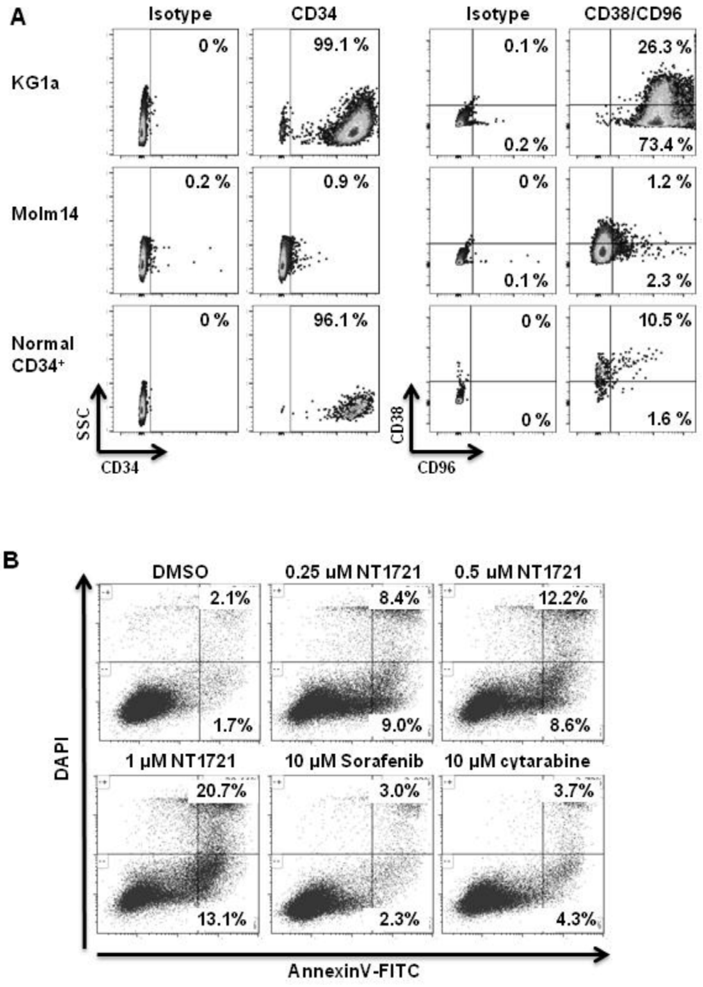

Supplementary Figure S1: Apoptosis induction in leukemic stem-like KG1a cells. (**A**) FACS analysis of the cell surface expression of CD34, CD38 and CD96 in KG1a, Molm14 and normal CD34$^+$ bone marrow cells. (**B**) KG1a cells were treated with NT1721, sorafenib or cytarabine for 48 h, stained with Annexin V and subjected to FACS analysis.

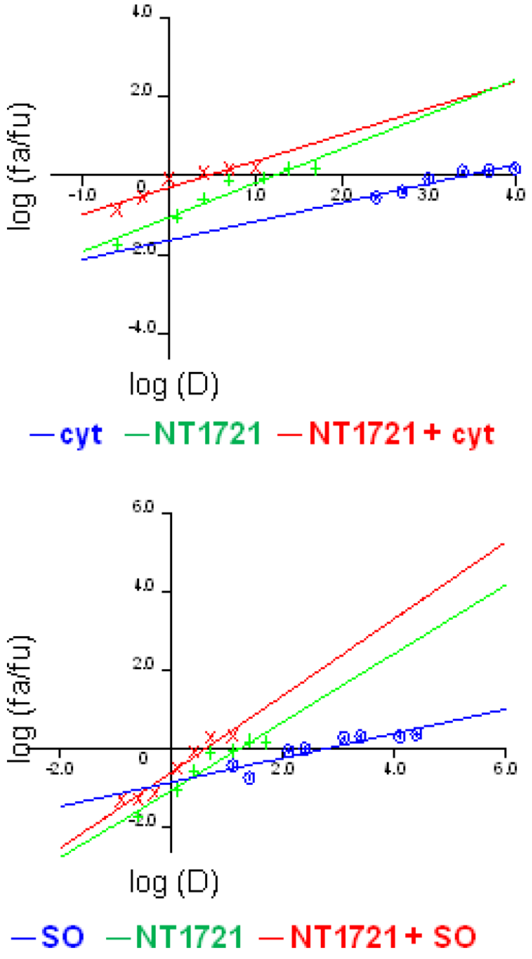

Supplementary Figure S2: Calculation of the combination index (CI) using Calcusyn software. Molm14 cells were treated with various concentration of the single agents (NT1721, cytarabine (cyt), sorafenib (SO)) or drug combinations for 48 h. The drug ratios were 1:100 and 1:10 for the NT1721:cyt and NT1721:SO combination, respectively. Data from the MTS cell viability assay were used to determine the CI.

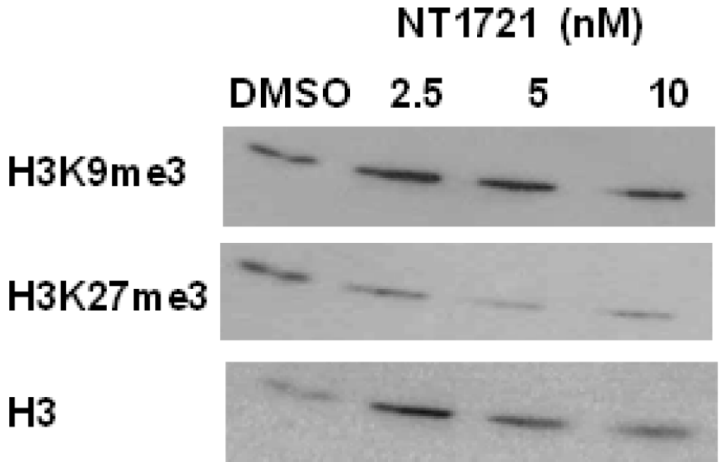

Supplementary Figure S3: Effect of NT1721 on global H3K9me3 and H3K27me3 levels. Molm14 cells treated with NT1721 for 48 h were subjected to Western blotting. The blot was stripped after detection of H3K9me3, probed with an H3K27me3 antibody, stripped and probed with an antibody detecting total H3.

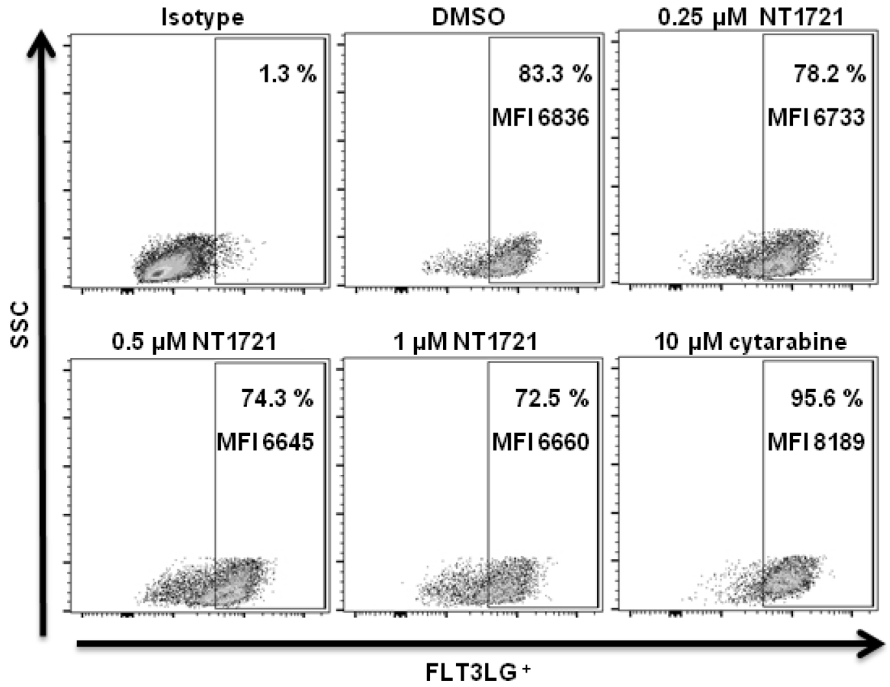

Supplementary Figure S4: NT1721 decreases FLT3LG expression in leukemic stem-like cells. KG1a cells were treated with the indicated concentrations of NT1721 or cytarabine. Treatment with NT1721 decreased FLT3LG expression in a dose-dependent manner while cytarabine increased the expression of FLT3LG (as measured by the increase in the mean fluorescence (MFI) and % positive cells).

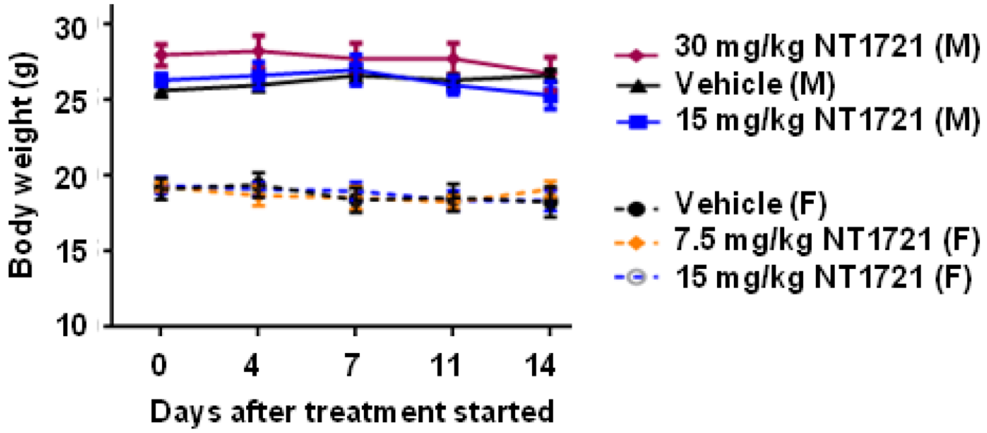

Supplementary Figure S5: Groups of NSG mice ($n \geq 3$) were treated with NT1721 as indicated. Their body weight was determined twice a week.
